# Evolutionary Optimization of Machining Parameters Based on Surface Roughness in End Milling of Hot Rolled Steel

**DOI:** 10.3390/ma14195494

**Published:** 2021-09-23

**Authors:** Issam Abu-Mahfouz, Amit Banerjee, Esfakur Rahman

**Affiliations:** School of Science, Engineering and Technology, Penn State Harrisburg, Middletown, PA 17057, USA; aub25@psu.edu (A.B.); aer15@psu.edu (E.R.)

**Keywords:** differential evolution, end milling, evolutionary algorithm, genetic algorithm, particle swarm optimization, surface roughness, vibration

## Abstract

Surface roughness measurements of machined parts are usually performed off-line after the completion of the machining operation. The objective of this work is to develop a surface roughness prediction method based on the processing of vibration signals during steel end milling operation performed on a vertical CNC machining center. The milling cuts were run under varying conditions (such as the spindle speed, feed rate, and depth of cut). This is a first step in the attempt to develop an online milling process monitoring system. The study presented here involves the analysis of vibration signals using statistical time parameters, frequency spectrum, and time-frequency wavelet decomposition. The analysis resulted in the extraction of 245 features that were used in the evolutionary optimization study to determine optimal cutting conditions based on the measured surface roughness of the milled specimen. Three feature selection methods were used to reduce the extracted feature set to smaller subsets, followed by binarization using two binarization methods. Three evolutionary algorithms—a genetic algorithm, particle swarm optimization and two variants, differential evolution and one of its variants, have been used to identify features that relate to the “best” surface finish measurements. These optimal features can then be related to cutting conditions (cutting speed, feed rate, and axial depth of cut). It is shown that the differential evolution and its variant performed better than the particle swarm optimization and its variants, and both differential evolution and particle swarm optimization perform better than the canonical genetic algorithm. Significant differences are found in the feature selection methods too, but no difference in performance was found between the two binarization methods.

## 1. Introduction

Surface roughness is an important quality characteristic for machined mechanical parts. Recent advancements in automated manufacturing and the increasing implementation of advanced manufacturing necessitates the use of nonintrusive surface roughness monitoring and predictive systems in machining operations. These systems are supported by modern artificial intelligence (AI) and machine learning (ML) techniques. In the past two decades, there has been a growing number of studies with focus on metal cutting parameters optimization and surface roughness prediction using AI techniques. The following is a brief review of the application of AI methods for surface roughness condition monitoring and optimization in end milling of steel parts.

A neural-fuzzy-based approach for in-process surface roughness recognition (ISRR) using vibration signals and cutting conditions is described in [1]. The support vector machine (SVM) algorithm is proposed in [2] to capture characteristics of surface roughness. It is then incorporated in a particle swarm optimization (PSO) algorithm to find optimum milling process parameters. A surface roughness model based on the back-propagation artificial neural network (BPANN) along with a genetic algorithm (GA) was used to optimize the cutting parameters in order to lower surface roughness [3]. In [4], two empirical models—one for cutting forces and another for surface roughness—were established with the help of ANOVA and range analysis methods. It was indicated that a linear model was a best fit for the variation of cutting forces while a quadratic model best described the variation of surface roughness. A review of the studies and investigation on the application of artificial neural networks (ANN) in the milling process for a period (1999–2010) was presented in [5]. Taguchi’s signal-to-noise ratio, L25 array, and ANOVA methods were employed in [6] to determine the effects of the spindle speed, feed rate, and depth of cut on the surface roughness in end milling of hardened AISI D2 steel. The results showed the spindle speed to be the most influencing parameters. In [7], singular spectrum analysis (SSA) is applied to investigate the relationship between cutting tool vibration and surface roughness in the precision end-milling process of hardened steel SCM440. Various architectures of the BPANN and the radial basis function neural network (RBFANN) have been investigated for surface roughness prediction [8]. A multi-criteria genetic algorithm (GA) optimization approach to minimize poor surface roughness and minimize electrical energy consumption during machining of AISI D2 steel was developed in [9]. The response surface methodology (RSM) has been used to develop mathematical models for predicting surface finish, tool vibration, and tool wear with different combinations of cutting parameters during end milling of EN-31 tool steel [10]. The experimental results show that the feed rate is the most dominating parameter. Various artificial neural network types are evaluated for the prediction of surface roughness [11]. The propagation Levenberg–Marquardt algorithm, back propagation Bayesian algorithm, and radial basis function neural networks are examined. An in-process intelligent neural-fuzzy surface roughness prediction and decision-making (INF-SRM) system for an end milling operation was developed in [12]. A Grey online modeling surface roughness monitoring (GOMSRM) system was proposed in [13]. This method utilized the Grey theory GM(1, N) with bilateral best-fit method with no training required. It was shown that the GOMSRM system had better accuracy with fewer samples for modeling than a neural network model. In [14], three models—fast Fourier transform long short-term memory network (FFT-LSTM), fast Fourier transform-deep neural networks (FFT-DNN), and one-dimensional convolutional neural network (1-D CNN)—are used to explore the prediction of surface roughness. Based on this experimental study, the FFT-LSTM or 1-D CNN is recommended to develop an intelligent system for surface roughness prediction based on vibration signals. Prediction models were developed using multiple regression analysis and an artificial neural network (ANN) modeling approach. The surface roughness and machining vibration levels were modeled with nonlinear quadratic forms based on the cutting parameters and their interactions through multiple regression analysis methods [15]. Analysis of variance (ANOVA) was employed to determine the significance of cutting parameters on surface roughness. The comparison between the prediction performance of the multiple regression and neural network-based models reveal that the ANN models achieve higher prediction accuracy for all training compared with regression models. An experimental model for estimating the surface roughness using artificial neural networks (ANN) and response surface methodology (RSM) has been improved for the dry milling of AA6061 alloy [16]. Analysis of variance (ANOVA) method was also used to study the influence of the cutting parameters on surface roughness. For this study, ANOVA results showed that the depth of cut is the most effective parameter on surface roughness. 

This study demonstrates the use of evolutionary algorithms for the optimization of steel end milling based on surface roughness and using process features extracted from vibration measurements. The remaining sections of the paper are organized as follows: Section 2 describes the experimental setup for vibration measurements and the processing of the acquired signals; in Section 3, the evolutional algorithms used in this study are described; the results and discussions are presented in Section 4 followed by the conclusions in Section 5. 

## 2. Experiment Setup and Feature Extraction

**Figure 1:**
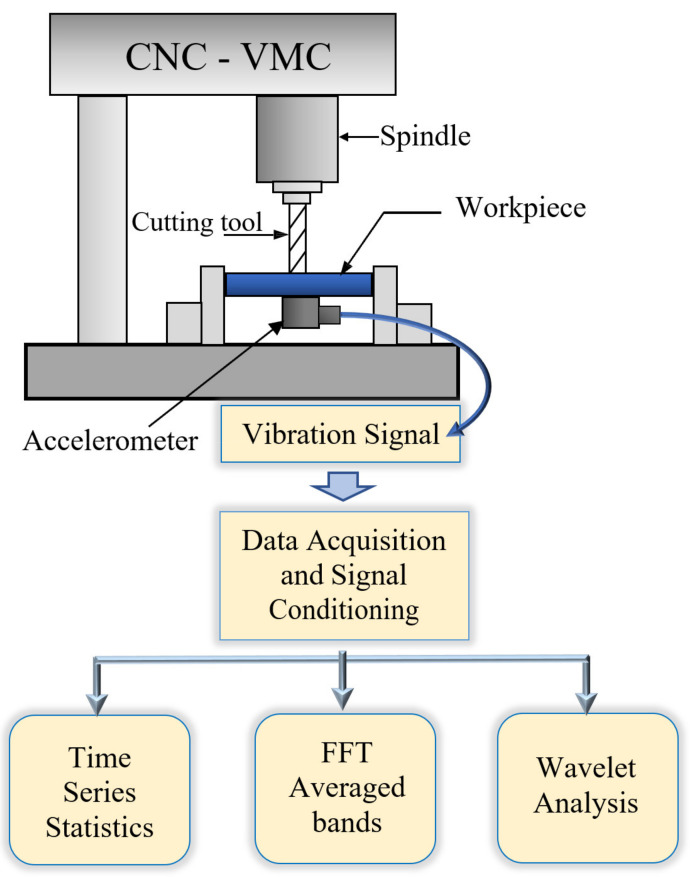
Schematic diagram of the end milling experimental set-up.

**Figure 2:**
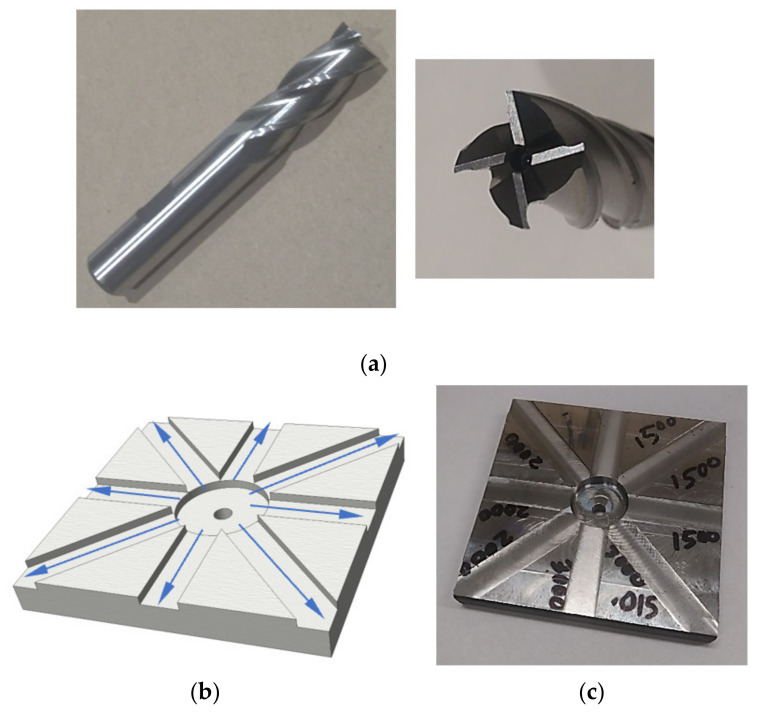
(**a**) End milling cutter, (**b**) milling paths, and (**c**) machined workpiece sample.

Figure 1 shows a schematic of the experimental setup for the end milling test. Several hot rolled low carbon steel (AISI A36) plates measuring 12.7 mm × 100 mm × 100 mm were used for this research work. Appendix A includes data on the composition (Table A1), mechanical properties (Table A2), and physical properties (Table A3) of the hot rolled low carbon steel (AISI A36). A 4-fluted helical uncoated carbide end mill cutter with diameter (*D* = 12.7 mm), shown in Figure 2a, was used in all tests. All tests were run on a 3-axis Hass minimill CNC vertical milling machine with a maximum spindle speed of 5000 rpm. An ICP 607A61 accelerometer mounted on the workpiece was used for measuring vibration in this test (with sensitivity of 100 mV/g and a frequency band of 0.5 to 10 kHz). The sensor was mounted on the center of the workpiece and cutting paths of equal length radiating from the center were machined as shown in Figure 2b. For each set of milling process parameter combinations, the vibration signals were sampled at 30 kHz for a duration of 3 s each and then recorded via a National Instruments USB-6211 data acquisition device. The sampling rate was chosen at three times higher than the maximum frequency of the accelerometer to allow for high frequency components within the signal to be captured. The surface roughness parameter (*Ra*), in µm, was selected as the measure for the surface roughness quality state in this study, measured using the Handysurf E-35A for each run. *Ra* was measured along the direction of cut (i.e., feed direction) and averaged over several milling runs for each parameter combination. The surface roughness parameter *Ra* is the most common parameter used to determine surface roughness and one of the common industry practices. Recently, 3D surface roughness data have been determined and can provide a thorough examination of a surface if needed. However, this process is costly and time consuming. On the other hand, the measurement of the *Ra* value is quick and can be completed on site. Adamczak et al. [17] performed a comparative study of 2D (*Ra*) and 3D surface roughness of bearing raceways on the vibration level and concluded that the *Ra* value provided better correlation with the vibration signal and the analysis of the 2D profile was adequate. Hence, the 3D surface analysis could be appropriate on a case-by-case basis. The focus of this investigation was to demonstrate the use of evolutionary algorithms for the optimization of surface roughness. Therefore, the most common industry practice was chosen to measure the surface roughness parameter.

Table 1 shows the averaged surface roughness data for all combinations of end milling parameters used in this work. The surface cutting speed *V_c_* in meters per minute (m/min) and the spindle speed (*N*) in revolutions per minute (RPM) are both presented. In this experiment, the spindle speed has been set and the surface sitting speed has been calculated as *V_c_* = *πDN*. Additionally, the feed rate *f,* presented in millimeters per tooth (mm/tooth), was calculated from the machine feed rate *V_f_* in millimeters per minute (mm/min) using the relation *f* = *V_f_*/*n_t_N*, where *n_t_* = 4 is the number of cutter teeth. The axial depth of cut (ap) is listed in mm and the radial depth of cut (ae) is fixed at 12.5 mm since the full cutter diameter is engaged during milling.

Statistical features were extracted from the raw time domain signals and from the extracted approximations and details signals using the Coiflet wavelet transform as described in [18]. The root-mean-square (RMS) is calculated by:

(1) $$\mathrm{RMS}=\sqrt{\frac{1}{N}\sum_{i=1}^{N}x_{i}^{2}}$$

where *x_i_* is the data sample and *N* is the number of data points in the interval of interest. The RMS value is an indication of the amount of energy in the vibration signal. The crest factor is defined as the ratio of the peak value to the root-mean-square (RMS) value of the vibration signal in this test and is calculated by:

Crest factor = Xpeak/RMS(2)

The variance is a statistical indicator of the data spread from the mean and is calculated as the average of the squared deviations in a data set from its mean value $\bar{x}=\frac{1}{N}\sum_{i=1}^{N}x_{i}$ as given by:

(3) $$\sigma^{2}=\frac{1}{N-1}\sum_{i=1}^{N}{\left(x_{i}-\bar{x}\right)}^{2}$$

Kurtosis is a measure of the frequency of occurrence of major peaks or outliers in the data and is therefore a representation of the statistical distribution of the amplitude of the signal. Kurtosis is defined as the fourth moment of the probability density curve about the mean and is given by:

(4) $$K=\frac{1}{N}\sum_{i=1}^{N}\frac{{\left(x_{i}-\bar{x}\right)}^{4}}{\sigma^{4}}$$

Skewness is a measure of symmetry or lack of symmetry of the data distribution about the mean. The skewness of a normal distribution is zero and the skewness of any symmetrical distribution about the mean is close to zero. The skewness is calculated as:

(5) $$S=\frac{1}{N}\sum_{i=1}^{N}\frac{{\left(x_{i}-\bar{x}\right)}^{3}}{\sigma^{3}}$$

In addition to the statistical quantities, 32 averaged frequency bands of the FFT (fast Fourier transform) of each signal segment and 64 averaged scalograms of the wavelet scalogram using the Coiflet and the Mexican Hat wavelets were taken as additional features in this study.

## 3. Methods

The problem of identifying optimal cutting conditions is cast as an optimization problem and solved using evolutionary computational techniques. A total of 245 features were extracted from the raw data (see Table 2 for description). Some of the features are highly correlated to other features and therefore by themselves have low discriminatory power. Reduced subsets of uncorrelated features are created by employing feature selection process. In a related work [19], we used a supervised recursive feature selection (RFE) method which produced the smallest subsets of features [20]. We also used a modified version of the Relief feature selection method called ReliefF [21]. Both RFE and ReliefF are supervised techniques employing class labels from surface roughness data. While RFE is a wrapper method, ReliefF is a filter method. In this paper, both RFE and ReliefF are used to produce reduced feature subsets using the Feature Selection Library (FSLib 2018), a publicly available MATLAB library for feature selection [22]. For comparison, 16 features are also used (kurtosis, skewness, rms, and crest factors of the top-two coefficients for wavelet approximations and details). The feature set will be referred to as the Statistical Features of Approximations and Details (SFAD) for the remainder of this paper. The original features and the reduced subsets are shown in Table 2. Before feature selection, all feature values within each type are normalized in the range 0–1. 

### 3.1. Genotype and Candidate Fitness

The use of binary encoding is preferred for its many advantages (in reproduction and exploration of solution spaces) in evolutionary algorithms. The normalized features after feature selection are binarized using two techniques—binarization by k-means clustering (BKMC) and binarization across multiple scales (BASC) [23]. The reduced feature set can then be represented as a binary-encoded string. The SFAD feature set is a binary string of length 16, while the RFE feature set is a binary string of length 15 and the ReliefF feature set is a binary string of length 26. They can be thought of as genetic expression with a high/low or an on/off scale (high/on: 1, low/off: 0). Each binary string will have a surface roughness label associated with it (as determined experimentally). The fitness of a binary string will correspond to its measured surface roughness, which will be used as its fitness value for selection during reproduction in the evolutionary algorithms. Due to effects of binarization and a limited experimental dataset, two or more individuals in a population could have the same binary-coded genotype but different surface roughness (and therefore correspond to different cutting conditions). We use the average value of the surface roughness as the fitness value of such individuals. For example, in the SFAD feature set, there are 65536 possible binary individuals; however, there are only 45 combinations for which surface roughness measurements are known. In case an individual in an evolutionary population has no associated fitness value, we use the Euclidean distance measure to find the nearest individual for which a surface roughness measure is known and assign that surface roughness value to the individual in question. There are multiple combinations of cutting conditions that provide good surface finish (small values of *R_a_*); therefore, the prediction problem can be used to suggest optimal cutting optimizations, i.e., the cutting speed and feed rate for a given axial depth of cut. The good surface finish is not always the smallest. The current study was not specific to any application, rather to report a general trend of surface roughness based on different milling parameter. Therefore, it was taken into consideration that as the *Ra* value decreases, the surface finish improves, and vice versa.

### 3.2. Evolutionary Algorithms

Evolutionary algorithms are population-based metaheuristic optimization algorithms used to solve complex optimization problems. The problem of identifying optimal cutting parameters that minimize surface roughness, a population of potential solutions are evolved starting from random for a fixed number of generations. The evolutionary process is guided by fitness—in this case, minimizing surface roughness. The potential solutions in the population are binary-encoded strings and are representations of the reduced feature sets. In this paper, a genetic algorithm with tournament selection, three particle swarm optimization algorithms and two differential evolution algorithms are used as evolutionary algorithms.

#### 3.2.1. Genetic Algorithm (GA)

The genetic algorithm used in this paper is described below:

(1) A random population of *P* individuals is initialized; each individual is a binary string of size corresponding to the size of the reduced feature set. Parameters such as the number of generations, *G*; and probabilities of mutation and crossover, *p_m_* and *p_c_*, respectively, are predetermined. Generation counter is initialized to 0. Values of these parameters can be found in Table 3.
(2) A mating pool is created using a tournament selector operator of size 2; two individuals are picked at random from the parent population and the one with the higher fitness (lower surface roughness value) is inserted into the mating pool. The size of the pool is the same as the population size.
(3) A pair of individuals is then picked sequentially from the mating pool and two offspring individuals created using a single point recombination operator with bit-wise mutation with probabilities *p_c_* and *p_m_*.
(4) The offspring population is merged with the parent population and the combined population is ranked according to individual fitness. The top half of the population is retained as the next generation.
(5) Generation counter is incremented, and steps 2–5 are repeated until generation counter = *G*.

#### 3.2.2. Particle Swarm Optimization (PSO)

PSO is a population-based stochastic optimization technique inspired by the swarm flocking behavior in nature [24]. A population of potential solutions is first initialized and updated every generation by following two “best” values—the first best value is the best location an individual has achieved till the present generation, also called personal best, and the other best value is the best location any individual has achieved till that generation, also called global best. An individual’s location is updated by first updating their velocity based on their current location in the search space, the personal best, and the global best. This location is then updated based on the newly updated velocity. At generation *i*, let the current position and velocity of individual *j* be ***p**_j_*(*i*) and ***v**_j_*(*i*), respectively. Let individual *j*’s personal best position be ***b**_j_* and let the global best of the entire population be ***b***. In the standard PSO (SPSO), the position and velocity of *j* are then updated in generation *i* + 1 as:

(6) $$v_{j}(i+1)=\omega (i)\;v_{j}(i)+c_{1}r_{1}[b_{j}-p_{j}(i)]+c_{2}r_{2}[b-p_{j}(i)]$$

(7) $$p_{j}(i+1)=p_{j}(i)+v_{j}(i+1)$$

Here, *ω*(*i*) is the *inertial weight* at the *i*th generation and determines the contribution of the previous velocity of an individual to its current one; *r*_1_ and *r*_2_ are uniformly distributed random variables in the range (0,1); *c*_1_ and *c*_2_ are constant parameters called *acceleration coefficients*, which define the magnitude of the influence of individual velocity in the direction of local and global optima, respectively. In this paper, the inertial weight and acceleration factors are linearly varied between fixed values at start and finish (see Table 3). For an implementation over *G* generations (1 ≤ *i* ≤ *G*), the inertial weight and the acceleration factors at generation *i* are given by:

(8) $$\omega (i)=[\omega (0)-\omega (G)]\frac{G-i}{G}+\omega (G)$$

(9) $$c_{k}(i)=[c_{k}(0)-c_{k}(G)]\frac{G-i}{G}+c_{k}(G);\;k=1,2$$

Variants of the standard PSO use various definitions and bounds for the acceleration coefficients and inertial weights. In this paper, a benchmarked variant of the PSO called the standard PSO-2011 or SPSO [25], the adaptive PSO or APSO [26], and the comprehensive learning PSO or CLPSO [27] are used.

#### 3.2.3. Differential Evolution (DE)

DE algorithms are population-based metaheuristic optimization algorithms which, unlike genetic algorithms, were specifically designed to work with real-valued strings. The algorithms use a recombination operator called the differential operator to create new candidate solutions [28]. This operator is a combination of mutation and crossover operators. The operator picks two candidates from the current generation and uses the difference between the two as the source of variation for a third candidate called the target vector. A mutant vector is created as:

(10) $$m_{j}(i+1)=r_{1}(i)+F[r_{2}(i)-r_{3}(i)]$$

Here, $r_{1}(i)$ is the target vector, $\left[r_{2}(i)-r_{3}(i)\right]$ is the difference between two randomly selected candidates, and *F* is the scale factor that controls the scale of the differential variation. The trial vector is defined component-wise as a binomial crossover operator,

(11) $$t_{jk}(i+1)=\left\{\begin{array}{cc} m_{jk}(i+1) & \mathrm{if}\;\eta \leq p_{c}\;\mathrm{or}\;k=\mathrm{rand}\_\mathrm{int}(1,N)\\ p_{jk}(i) & \mathrm{otherwise} \end{array}\right.$$

where *η* is a random number in (0,1) and *p_c_* is the probability of crossover. In (11) $t_{jk}$, $m_{jk}$, and $p_{jk}$ refer to the *j*th candidate’s *k*th bit in the target, mutant, and candidate vector, respectively. The random integer *rand_int* is an integer in the range (1, *N*) and is used to ensure that at least one mutant vector bit goes into constructing the trial vector. A tournament selection operator such as a one-to-one comparison operator is used to evolve the candidate vector. The trial vector replaces the candidate vector if it is better, as measured by the fitness function as:

(12) $$p_{j}(i+1)=\left\{\begin{array}{cc} t_{j}(i+1) & \mathrm{if}\;f(t_{j}(i+1)<f(p_{j}(i))\\ p_{j}(i) & \mathrm{otherwise} \end{array}\right.$$

The performance of DE depends on the strategy used to generate trial vectors and how well the control parameters are tuned to solve the specific problem. A variant of the differential evolution algorithm called the composite differential evolution (CoDE) [29] combines the three most popular trial candidate generation strategies with three control parameter settings, which are used in a random manner to generate trial candidates. It has a simple structure and is easy to implement. The three trial vector generation strategies for CoDE used in this paper are rand/1, best/1, and current-to-best/2. The parameters discussed in this section for the three algorithms and their variants are presented in Table 3. Some of these parameters were tuned in a test environment prior to deployment, while other parameters were assigned based on information in literature.

## 4. Results

**Figure 3:**
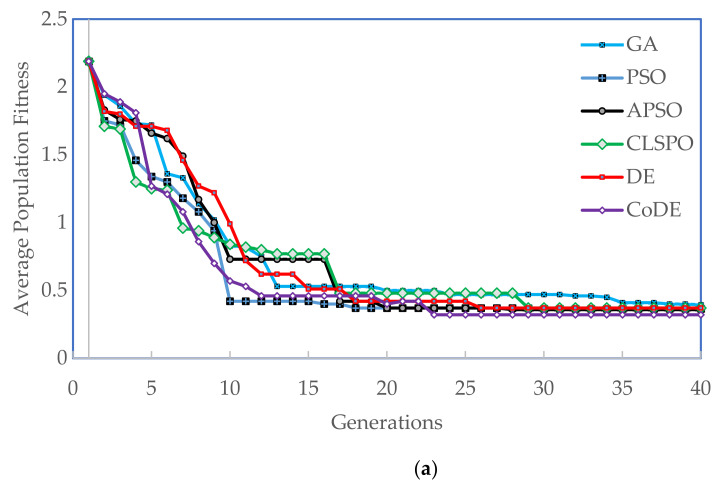

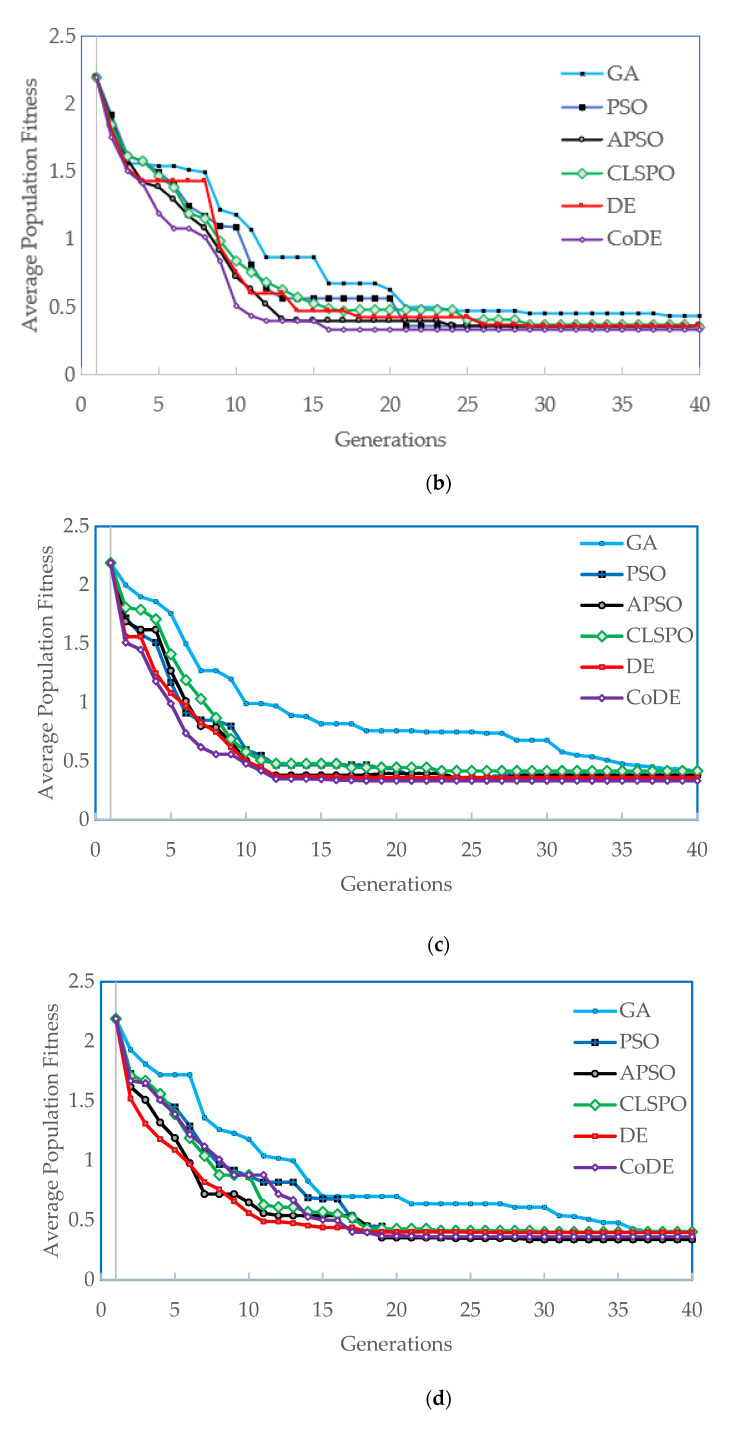
(**a**) Average population fitness as a function of the number of generations for ReliefF-BKMC. (**b**) Average population fitness as a function of the number of generations for RFE-BKMC. (**c**) Average population fitness as a function of the number of generations for ReliefF-BASC. (**d**) Average population fitness as a function of the number of generations for RFE-BASC.

The parameters of the algorithms used are provided in Table 3. The algorithms are run for a total of 10 times each for every feature set and the results presented in this section are an average of the 10 runs. The individual with the highest fitness is chosen as the solution of a particular run. Performance of the evolutionary algorithms is evaluated based on (1) maximum individual fitness in the terminating generation, and (2) average fitness of the terminating population. The maximum individual fitness for the six evolutionary algorithms with the three feature sets and two binarization schemes is tabulated in Table 4 and the average fitness of the terminating population is presented in Table 5. The convergence of the algorithms to the best individual is also tracked. The same best solution as listed in Table 4 is tracked and the earliest generation to feature the best solution is reported. This is reported in Table 6. As can be seen, the best solution is found consistently at earlier generations with DE and CoDE compared to PSO and its variants. The GA does not converge in many of the test cases. The quality of the solution is also significantly better using DE and CoDE algorithms. Among the reduced feature sets, the RFE performed marginally better than ReliefF and both RFE and ReliefF were significantly better than SFAD. Among the two binarization methods, there was no easily discernable difference between BKMC and BASC, although the BKMC produced solutions that had better fitness, BASC converged sooner in most cases to the best solution compared to BKMC binarized feature sets. The average fitness of the populations starting from the same initial population is tracked till the termination generation (*G* = 40) in Figure 3. ReliefF and RFE feature sets for both binarizations are shown. APSO, CLPSO, and CoDe converge quicker to a stable final population while the canonical versions of PSO and DE are slower to converge; in some cases, the GA did not converge to a stable solution (compared to the best performing algorithm that converged in half the time).

## 5. Discussion

All the evolutionary algorithms tested here are able to converge to a reasonable terminating population; the best individual in the population is very close to the average fitness (with some exceptions). The solutions in the converged final population encode for favorable values of surface finish (low surface roughness *R_a_*). In most cases, that might be desirable; however, in some cases, the intention might be create a rough surface. The framework presented here can be used to converge to a population of individuals with high values of *R_a_* by changing the fitness function to reward high values of *R_a_* instead of low values. Although, the individual with the best fitness is reported here (in Table 4), in practice, the average population fitness of the terminating population and the average change in fitness between the initial and the final populations are of more interest. The individuals in the terminating population can be decoded and their cutting parameters (cutting speed, feed rate, and axial depth of cut (ap)) can be found. It is not the objective of the paper to locate the absolute values of the “best” cutting parameters but instead to provide a framework to find a population of decoded parameters that correspond to a set of preferred cutting parameters (instead a single “best” parameter set).

## 6. Conclusions

In this paper, we have demonstrated the applicability of evolutionary algorithms to optimize cutting conditions for milling. The cutting conditions are optimized indirectly by driving the evolutionary algorithms towards solutions in a reduced feature space based on measured values of surface roughness. The feature space includes raw time series statistical quantities, frequency spectrum bands, and time-frequency wavelets decompositions and their statistical parameters such as the kurtosis, skewness, and RMS values. Three feature selection techniques followed by two binarization methods on the reduced feature sets are used to create candidate solutions for the evolutionary algorithms. All algorithms tested here—genetic algorithm (GA), particle swarm optimization (PSO) and its two variants (APSO and CLPSO), and a differential evolution (DE) algorithm and its variant (CoDE)—were initialized with the same random initial population and then evolved using a fitness function based on measured surface roughness values. The populations are shown to converge quickly to stable populations with low average and minimum (optimal) values of surface roughness. DE and CoDE are also shown to be more effective than PSO, APSO, and CLPSO. All five of these algorithms work better than the canonical GA. However, the GA is easier to tune and therefore remains a viable option. The framework presented in this paper can be easily scaled up, either by using a model-driven fitness function or if more experimental data were available. The procedure can also be applied to other machining processes where feature-rich sensor data are collected in real time during machining. In future work, we will use statistical measures to compare significance of results and ascertain if any feature selection technique, binarization method, or evolutionary algorithms are statistically superior to other techniques and methods used in this paper.

## Figures and Tables

**Table 1:** Average surface roughness measures *Ra* for the steel milling test.

**Axial depth of cut (ap) = 0.381 mm**									
Speed	*N* = 1500 rpm			*N* = 2000 rpm			*N* = 3000 rpm		
	*V_c_* = 59.847 m/min			*V_c_* = 79.796 m/min			*V_c_* = 119.695 m/min		
*f* (mm/tooth) *V_f_* (mm/min)	0.0127 76.2	0.0169 101.6	0.0212 127.0	0.0095 76.2	0.0127 101.6	0.0159 127.0	0.0085 101.6	0.0106 127.0	0.0127 152.4
*Ra* (µm)	0.416	0.302	0.326	0.328	0.304	0.454	0.364	0.314	0.414
Speed	*N* = 4000 rpm			*N* = 5000 rpm			*-*		
	*V_c_* = 159.593 m/min			*V_c_* = 199.491 m/min			*-*		
*f* (mm/tooth) *V_f_* (mm/min)	0.0079 127.0	0.0095 152.4	0.0111 177.8	0.0076 152.4	0.0089 177.8	0.0102 203.2	*-*	*-*	*-*
*Ra* (µm)	0.392	0.282	0.328	0.302	0.424	0.448	*-*	*-*	*-*
**Axial depth of cut (ap) = 0.762 mm**									
Speed	*N* = 1500 rpm			*N* = 2000 rpm			*N* = 3000 rpm		
	*V_c_* = 59.847 m/min			*V_c_* = 79.796 m/min			*V_c_* = 119.695 m/min		
*f* (mm/tooth) *V_f_* (mm/min)	0.0127 76.2	0.0169 101.6	0.0212 127.0	0.0095 76.2	0.0127 101.6	0.0159 127.0	0.0085 101.6	0.0106 127.0	0.0127 152.4
*Ra* (µm)	0.93	0.852	0.558	0.64	0.896	0.902	0.494	0.418	0.482
Speed	*N* = 4000 rpm			*N* = 5000 rpm			*-*		
	*V_c_* = 159.593 m/min			*V_c_* = 199.491 m/min			*-*		
*f* (mm/tooth) *V_f_* (mm/min)	0.0079 127.0	0.0095 152.4	0.0111 177.8	0.0076 152.4	0.0089 177.8	0.0102 203.2	*-*	*-*	*-*
*Ra* (µm)	0.834	0.678	0.384	0.864	1.048	1.638	*-*	*-*	*-*
**Axial depth of cut (ap) = 1.524 mm**									
Speed	*N* = 1500 rpm			*N* = 2000 rpm			*N* = 3000 rpm		
	*V_c_* = 59.847 m/min			*V_c_* = 79.796 m/min			*V_c_* = 119.695 m/min		
*f* (mm/tooth) *V_f_* (mm/min)	0.0127 76.2	0.0169 101.6	0.0212 127.0	0.0095 76.2	0.0127 101.6	0.0159 127.0	0.0085 101.6	0.0106 127.0	0.0127 152.4
*Ra* (µm)	1.186	0.814	0.726	0.596	0.838	0.868	1.34	1.108	1.01
Speed	*N* = 4000 rpm			*N* = 5000 rpm			*-*		
	*V_c_* = 159.593 m/min			*V_c_* = 199.491 m/min			*-*		
*f* (mm/tooth) *V_f_* (mm/min)	0.0079 127.0	0.0095 152.4	0.0111 177.8	0.0076 152.4	0.0089 177.8	0.0102 203.2	*-*	*-*	*-*
*Ra* (µm)	0.82	1.612	1.49	2.084	1.98	1.79	*-*	*-*	*-*

**Table 2:** Features extracted from vibrations data.

Feature Name	Original Size	ReliefF	RFE	SFAD
Fast Fourier Transform Averages	32	4	2	0
Mean of raw time series	1	1	1	0
Skewness of raw time series	1	1	0	0
Standard deviation of raw time series	1	0	1	0
Kurtosis of raw time series	1	0	0	0
Variance of raw time series	1	0	0	0
Mexican Hat coefficients	64	8	5	0
Coiflet wavelet coefficients	64	6	2	0
Kurtoses of wavelet approximations	10	1	1	2
Skewness of wavelet approximations	10	1	0	2
Kurtoses of wavelet details	10	1	0	2
Skewness of wavelet details	10	0	1	2
RMS of wavelet approximations	10	1	0	2
RMS of wavelet details	10	1	1	2
Crest factors of wavelet approximations	10	1	1	2
Crest factors of wavelet details	10	0	0	2
**Total**	**245**	**26**	**15**	**16**

**Table 3:** Parameters for the evolutionary algorithms.

	GA	PSO	APSO	CLPSO	DE	CoDE
Population size *P*	25	25	25	25	25	25
Max generations *G*	40	40	40	40	40	40
Crossover probability *p_m_*	0.9	-	-	-	0.9	0.9
Mutation probability *p_c_*	0.05	-	-	-	-	-
Inertial weight *ω* (start, end)	-	0.9, 0.4	0.9, *^1^	0.9, 0.4	-	-
Acceleration weight 1, *c*_1_ (start, end)	-	0.5, 2.5	2.0, *^2^	-	-	-
Acceleration weight 2, *c*_2_ (start, end)	-	2.5, 0.5	2.0, *^2^	-	-	-
Acceleration coefficient, *c*	-	-		1.5	-	-
Scale factor *F*	-	-	-		0.9	0.9

*^1^ Adaptive—monotonic with evolutionary factor *f*. *^2^ Adaptive—strategy 1 till generation 15, then strategy 2 till convergence [26].

**Table 4:** Maximum individual fitness (individuals with smallest surface roughness) in terminating generation.

		Evolutionary Algorithms					
Feature Sets	Binarization	GA	PSO	APSO	CLPSO	DE	CoDE
ReliefF	BKMC	0.375	0.326	0.302	0.318	0.298	0.292
ReliefF	BASC	0.392	0.338	0.322	0.329	0.309	0.301
RFE	BKMC	0.357	0.302	0.302	0.302	0.311	0.302
RFE	BASC	0.382	0.329	0.329	0.329	0.329	0.314
SFAD	BKMC	0.428	0.389	0.372	0.370	0.358	0.328
SFAD	BASC	0.440	0.402	0.398	0.382	0.369	0.364

**Table 5:** Average population fitness (average surface roughness) in terminating generation.

		Evolutionary Algorithms					
Feature Sets	Binarization	GA	PSO	APSO	CLPSO	DE	CoDE
ReliefF	BKMC	0.395	0.366	0.359	0.371	0.371	0.321
ReliefF	BASC	0.409	0.387	0.381	0.418	0.362	0.334
RFE	BKMC	0.432	0.353	0.357	0.352	0.361	0.331
RFE	BASC	0.408	0.344	0.340	0.402	0.398	0.362
SFAD	BKMC	0.488	0.431	0.421	0.479	0.388	0.377
SFAD	BASC	0.540	0.501	0.476	0.424	0.404	0.374

**Table 6:** Earliest generation to converge to best solution.

		Evolutionary Algorithms					
Feature Sets	Binarization	GA	PSO	APSO	CLPSO	DE	CoDE
ReliefF	BKMC	40 *	32	28	27	22	20
ReliefF	BASC	40 *	29	25	20	18	17
RFE	BKMC	38	26	22	24	23	22
RFE	BASC	37	28	29	28	26	23
SFAD	BKMC	40 *	31	30	29	27	27
SFAD	BASC	40 *	35	32	27	29	27

* does not converge.

## Data Availability

The data presented in this study are available on request from the corresponding author after obtaining permission of authorized person.

## Appendix A

Work piece: Hot rolled Low Carbon Steel (AISI A36).

**Table A1:** Composition of Hot rolled Low Carbon Steel (AISI A36).

Element	Weight %
Carbon (C)	0.26
Copper (Cu)	0.2
Manganese (Mn)	0.75
Phosphorous (P)	0.04 max
Sulphur (S)	0.05 max
Iron (Fe)	Balance

**Table A2:** Mechanical Properties of Hot rolled Low Carbon Steel (AISI A36).

Property	Value
Tensile Strength (annealed)	400–545 MPa
Ductility	22%
Hardness	140 BHN

**Table A3:** Physical Properties Hot rolled Low Carbon Steel (AISI A36).

Property	Value
Density, g/cm^3^	7.87
Melting point	1421–1465 °C
Thermal conductivity (W/m·K)	89.0 (20 °C)
Coefficient of thermal expansion, (10^−6^/K)	12.4 (20–100 °C)

## References

[1] Lou S.-J., Chen J.C. (1999). In-Process Surface Roughness Recognition (ISRR) System in End-Milling Operations. Int. J. Adv. Manuf. Technol..

[2] Chakguy P., Siwaporn K., Pisal Y. (2009). Optimal cutting condition determination for desired surface roughness in end milling. Int. J. Adv. Manuf. Technol..

[3] Öktem H. (2009). An integrated study of surface roughness for modelling and optimization of cutting parameters during end milling operation. Int. J. Adv. Manuf. Technol..

[4] Ding T., Zhang S., Wang Y., Zhu X. (2010). Empirical models and optimal cutting parameters for cutting forces and surface roughness in hard milling of AISI H13 steel. Int. J. Adv. Manuf. Technol..

[5] Al-Zubaidi S., Ghani J.A., Haron C.H.C. (2011). Application of ANN inMilling Process: A Review. Model. Simul. Eng..

[6] Zaidan A., Shammari M., Amwead K.A., Hadi A.S. Effect of Cutting Parameters on Surface Roughness When Milling Hardened AISI D2 Steel (56 HRC) Using Taguchi Techniques. Proceedings of the ASME 2012 International Mechanical Engineering Congress & Exposition IMECE2012.

[7] Chen C.C., Liu N.M., Chiang K.T., Chen H.L. (2012). Experimental investigation of tool vibration and surface roughness in the precision end-milling process using the singular spectrum analysis. Int. J. Adv. Manuf. Technol..

[8] Saric T., Simunovic G., Simunovic K. (2013). Use of Neural Networks in Prediction and Simulation of Steel Surface Roughness. Int. J. Simul. Model..

[9] Alrashdan A., Bataineh O., Shbool M. (2014). Multi-criteria end milling parameters optimization of AISI D2 steel using genetic algorithm. Int. J. Adv. Manuf. Technol..

[10] Bhogal S.S., Sindhu C., Dhami S.S., Pabla B.S. (2015). Minimization of Surface Roughness and Tool Vibration in CNC Milling Operation. J. Optim..

[11] Angelos P., Markopoulos S.G., Dimitrios E.M. (2016). On the use of back propagation and radial basis function neural networks in surface roughness prediction. J. Ind. Eng. Int..

[12] PoTsang B.H. (2016). An intelligent neural-fuzzy model for an in-process surface roughness monitoring system in end milling operations. J. Intell. Manuf..

[13] PoTsang B.H., Zang H.J., Lin Y.C. (2019). Development of a Grey online modeling surface roughness monitoring system in end milling operations. J. Intell. Manuf..

[14] Lin W.J., Lo S.H., Young H.T., Hung C.L. (2019). Evaluation of Deep Learning Neural Networks for Surface Roughness Prediction Using Vibration Signal Analysis. Appl. Sci..

[15] Lin Y.C., Wu K.D., Shih W.C., Hsu P.K., Hung J.P. (2020). Prediction of Surface Roughness Based on Cutting Parameters and Machining Vibration in End Milling Using Regression Method and Artificial Neural Network. Appl. Sci..

[16] Eser A., Ayyildiz E.A., Ayyildiz M., Kara F. (2021). Artificial Intelligence-Based Surface Roughness Estimation Modelling for Milling of AA6061 Alloy. Adv. Mater. Sci. Eng..

[17] Adamczak S., P Zmarzły P. (2019). Research of the influence of the 2D and 3D surface roughness parameters of bearing raceways on the vibration level. IOP Conf. Ser. J. Phys. Conf. Ser..

[18] Abu-Mahfouz I., El Ariss O., Rahman A.H.M.E., Banerjee A. (2017). Surface roughness prediction as a classification problem using support vector machine. Int. J. Adv. Manuf. Technol..

[19] Abu-Mahfouz I., Banerjee A., Rahman E. (2021). Evaluation of Clustering Techniques to Predict Surface Roughness during Turning of Stainless-Steel Using Vibration Signals. Materials.

[20] Guyon I., Weston J., Barnhill S., Vapnik V. (2002). Gene selection for cancer classification using support vector machines. Mach. Learn..

[21] Kononenko I., Simec E., Robnik-Sikonja M. (1997). Overcoming the myopia of indictive algorithms with RELIEFF. Appl. Intell..

[22] Roffo G. Feature Selection Library, MATLAB Central File Exchange. https://www.mathworks.com/matlabcentral/fileexchange/68210-feature-selection-library.

[23] Hopfensitz M., Mussel C., Wawra C., Maucher M., Kuhl M., Neumann H., Kestler H.A. (2012). Multiscale binarization of gene expression data for reconstructing Boolean networks. IEEE/ACM Trans. Comput. Biol. Bioinform..

[24] Kennedy J., Eberhart R. Particle swarm optimization. Proceedings of the IEEE International Conference on Neural Networks.

[25] Zambrano-Bigiarini M., Clerc M., Rojas R. Standard particle swarm optimization 2011 at CEC-2013: A baseline for future PSO improvements. Proceedings of the 2013 IEEE Congress on Evolutionary Computation.

[26] Zhan Z.H., Zhang J., Li Y., Chung H.S.H. (2009). Adaptive particle swarm optimization. IEEE Trans. Syst. Man Cybern. Part B Cybern..

[27] Liang J.J., Qin A.K., Suganthan P.N., Baskar S. (2006). Comprehensive learning particle swarm optimizer for global optimization of multimodal functions. IEEE Trans. Evol. Comput..

[28] Storn R., Price K. (1997). Differential Evolution—A simple and efficient heuristic for global optimization over continuous spaces. J. Glob. Opt..

[29] Wang Y., Cai A., Zhang Q. (2011). Differential evolution with composite trial vector generation strategies and control parameters. IEEE Trans. Evol. Comput..

